# Seed bank contributions and environmental filtering shape seasonal dynamics and restoration potential of submerged macrophytes in Baiyangdian Lake

**DOI:** 10.3389/fpls.2026.1830771

**Published:** 2026-05-08

**Authors:** Kai Mo, Chen Wang, Yu Jin, Zhan Shi, Yanzhe Tian, Lei Jin, Cunqi Liu

**Affiliations:** 1 College of Life Science, Hebei University, Baoding, China; 2 Field Scientific Observation and Research Station of Lake and Wetland Ecosystems in Baiyangdian Basin, College of Life Sciences, Hebei University, Baoding, China

**Keywords:** community assembly, recovery potential, seasonal dynamics, seed bank, submerged macrophytes

## Abstract

**Introduction:**

Submerged macrophytes are essential for maintaining the structure and functioning of shallow lake ecosystems. Seasonal environmental fluctuations strongly shape the structure and recovery capacity of submerged macrophyte communities.

**Methods:**

To evaluate seasonal recovery potential and environmental constraints in Baiyangdian Lake, we integrated species richness, biomass, community structure, β−diversity components, and assembly processes with water and sediment variables based on field surveys, seed bank germination experiments, and multivariate analyses.

**Results:**

The results showed that seed bank had the highest species richness and contained four unique species, whereas richness in submerged macrophyte communities declined steadily from spring to autumn. PERMANOVA revealed significant seasonal differences in community structure (R² = 0.1662, *P* = 0.001). β−diversity partitioning indicated that richness differences dominated community assembly in spring and summer, whereas species replacement became predominant in autumn. Deterministic processes dominated community assembly overall, with environmental filtering strongest in autumn. Key environmental drivers shifted seasonally: chlorophyll-a (*Chl.a*), ratio of secchi depth (SD) to water depth (WD), and sediment total phosphorus (STP) were significant in spring; STP was the sole significant driver in summer; and sediment organic matter (SOM) emerged as the primary driver in autumn. Using a decision-matrix framework that integrates recovery potential and environmental pressure, we classified site trajectories into five seasonal types, including continuous high-risk, seasonal improvement, cumulative stress, stable-resistant, and abrupt deterioration, each representing distinct ecological response pathways that highlight differences in resilience, stress accumulation, and management priorities across seasons.

**Discussion:**

These findings provide a process−based foundation for ecological risk diagnosis and support season−specific restoration and adaptive management in shallow lake ecosystems.

## 1 Introduction

Submerged macrophytes are indispensable components of shallow lake ecosystems, performing critical ecological functions. They stabilize sediments to reduce resuspension, release allelopathic substances that inhibit phytoplankton growth and improve water clarity (Madsen et al., 2001). They also provide spawning grounds, feeding habitats, and refuge for aquatic organisms, thereby sustaining biodiversity and food web integrity (Hilt et al., 2018). They regulate nutrient cycling by absorbing nitrogen and phosphorus from water and sediment, mitigating eutrophication and maintaining ecosystem stability (Dan et al., 2021; Hao et al., 2024; Human et al., 2015). Understanding the mechanisms driving community assembly, spatiotemporal dynamics, and natural recovery potential of submerged macrophytes has long been a central topic in aquatic plant ecology and restoration biology.

β-diversity partitioning and the quantification of deterministic versus stochastic processes are key tools to disentangle plant community assembly mechanisms. β-diversity decomposes community dissimilarity into species replacement and richness difference, while assembly analyses distinguish between deterministic (e.g., environmental filtering) and stochastic processes (e.g., dispersal limitation) (Baselga, 2010; Riddley et al., 2025; Powell et al., 2015). However, the seasonal dynamics of these assembly mechanisms remain insufficiently understood in submerged macrophyte communities.

The sediment seed bank is a critical natural reservoir for aquatic plant diversity maintenance and community recovery. It preserves propagules of native submerged macrophyte species, buffers against environmental disturbances, and provides the core germplasm for natural community restoration (An et al., 2022; Casanova, 2015). Seasonal fluctuations in environmental factors are key drivers of both submerged macrophyte growth and seed bank germination. Water-column variables such as chlorophyll-a (*Chl.a*) and water depth (WD) directly influence light availability, a critical determinant of submerged macrophyte photosynthesis and seedling establishment (Chambers and Prepas, 1990). Sediment properties, including sediment total phosphorus (STP) and sediment organic matter (SOM), regulate nutrient supply and sediment redox conditions. STP provides essential nutrients for plant growth and seed germination, whereas excessive SOM reduces sediment oxygen content, thereby inhibiting root growth and seed viability (Lu et al., 2021; Song et al., 2013). Previous studies on submerged macrophytes have primarily examined the seasonal dynamics of aboveground vegetation, whereas few have integrated seed bank potential with aboveground community assembly processes. In particular, how seasonal shifts in environmental drivers regulate the coupling between the seed bank and standing vegetation, and how this coupling influences community recovery potential, remain key knowledge gaps in aquatic plant ecology (Choi and Kim, 2022; Rohal et al., 2024).

Furthermore, a methodological disconnect persists between ecological monitoring and management action. Existing studies identify environmental drivers but rarely translate these findings into predictive frameworks that guide managers in determining where and when restoration is most likely to succeed, based on combined assessments of recovery potential and environmental pressure (Liu et al., 2023; Rybak et al., 2024; Yuan et al., 2007).

Shallow lakes in northern China (e.g., Baiyangdian Lake) experience strong seasonal fluctuations in temperature, hydrology, and nutrient conditions (Yu et al., 2023; Zhang et al., 2011), which impose intense and dynamic environmental filtering on submerged macrophyte communities (Li et al., 2017; Papastergiadou et al., 2010). Baiyangdian Lake is a core ecological conservation area for the Xiong’an New Area, and its submerged macrophyte communities have undergone significant degradation and restoration in recent decades (Han et al., 2022; Li et al., 2016). While some studies have described the spatial distribution of submerged macrophytes in this lake, the seasonal assembly mechanisms, β-diversity dynamics, and seed bank-mediated recovery potential of these communities have not been systematically quantified.

Here, we integrated seasonal field surveys, seed bank germination experiments, and multivariate statistical analyses to investigate the seasonal dynamics of submerged macrophyte communities in Baiyangdian Lake. We addressed three core scientific questions: (1) How do β−diversity components and community assembly processes of submerged macrophytes shift across seasons? (2) What are the key seasonal environmental drivers regulating community structure and assembly? (3) How can seed bank potential and environmental pressure be integrated to classify community recovery trajectories? Our findings advance the understanding of seasonal assembly mechanisms in submerged macrophyte communities and provide a mechanistic basis for conserving and restoring aquatic plant diversity in shallow lake ecosystems.

## 2 Materials and methods

### 2.1 Study area

Baiyangdian Lake (E 115°45′–116°26′, N 38°44′–39°00′), situated in the central North China Plain, is the largest shallow freshwater lake in northern China and a key component of the Xiong’an New Area ecological protection system (Wang et al., 2018; Zhao et al., 2011). The lake experiences a temperate continental monsoon climate with distinct seasonal variation: mean annual temperature of 12.7 °C, mean precipitation of 552 mm (mainly June–August), and pronounced autumn evaporation (September–November). It covers ~366 km², with an average depth of 1.5–3.0 m, and is characterized by complex hydrology (e.g., seasonal water-level fluctuations) and intense human disturbance (e.g., agricultural non-point source pollution, aquaculture).

### 2.2 Field sampling and analysis

**Figure 1:**
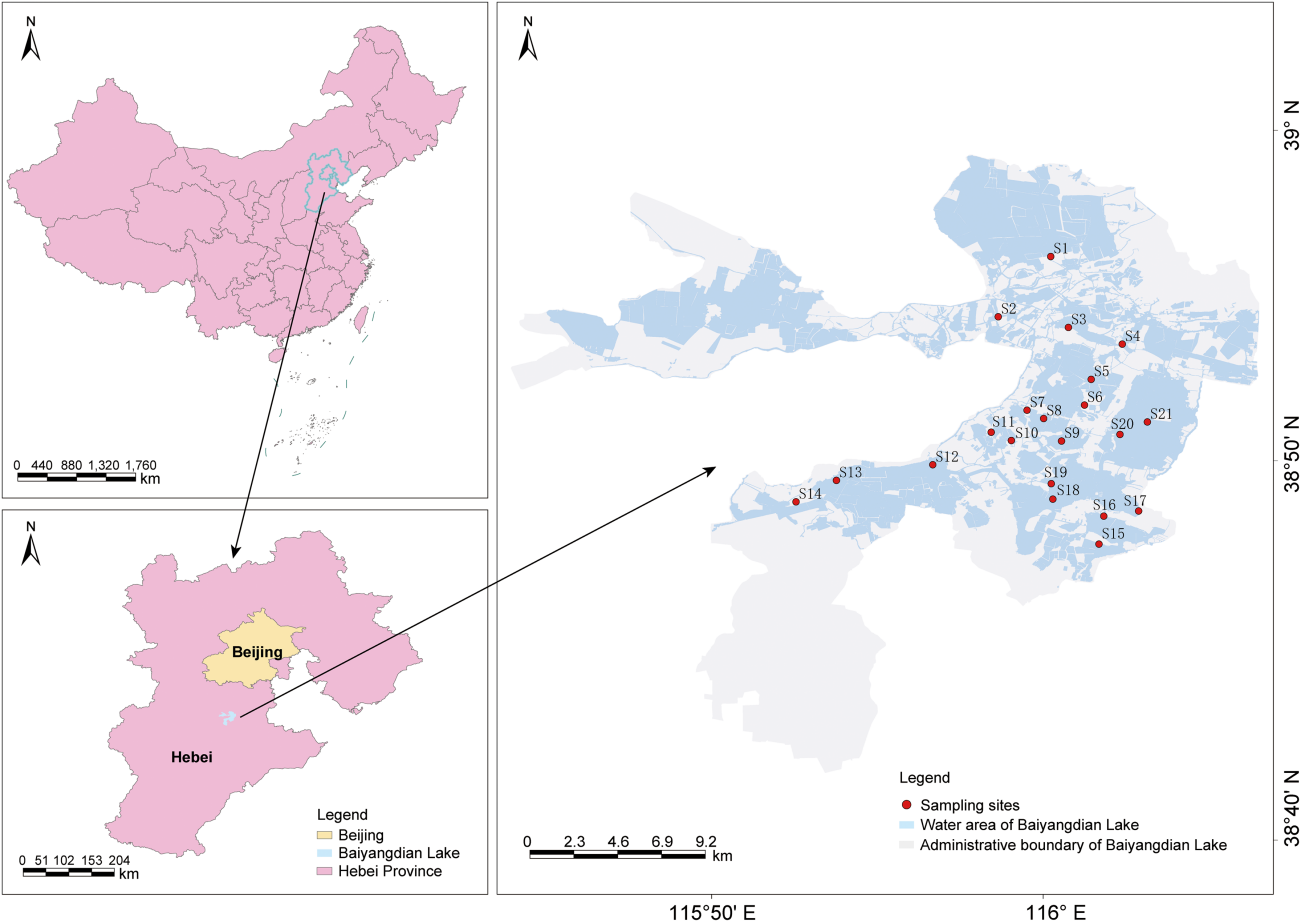
Location of the study area and sampling sites.

Field sampling was conducted in spring (April 2024), summer (July 2024), and autumn (October 2024) to represent key growth stages of submerged macrophytes. Twenty-one sampling sites were selected based on hydrological conditions and macrophyte distribution (**Figure 1**), covering open-water zones, nearshore areas, and regions with moderate anthropogenic influence such as low-intensity aquaculture. All sites were georeferenced using a handheld GPS (Garmin, USA) unit to ensure consistent resampling across seasons.

At each site, three replicate 1 × 1 m quadrats were established. Within each quadrat, all submerged macrophyte species were identified morphologically using taxonomic keys (Cook et al., 1974). The abundance (individuals per species) and coverage (%) of each species were recorded. Submerged macrophytes were collected, rinsed to remove sediment and epiphytes, weighed for fresh mass, and biomass was calculated.

Seed bank sampling was conducted concurrently in spring. Spring (April) represents the peak of seed bank density before the main germination period, which is a standard practice for assessing the potential seed bank for the upcoming growing season in temperate lakes (Thompson et al., 1997). Five replicate surface sediment (0–10 cm) samples were collected using a Peterson grab sampler and pooled into a composite sample at each site. Sediments were sieved through a 0.2 mm mesh to concentrate the samples. The concentrated sediment samples consisted of both sexual seeds and asexual vegetative propagules (e.g., tubers, turions). Germination trays were prepared with a 1 cm layer of heat−treated sand and a 3 cm layer of concentrated sediment, following widely applied germination methods. A total of 21 trays were placed in a greenhouse with adequate light and maintained under submerged conditions. The experiment continued until no new seedlings emerged, followed by an additional week of observation.

For water samples, three replicates were collected and stored in polyethylene bottles. Secchi depth (SD) was measured *in situ* using a black and white Secchi disk, and WD was recorded with a portable depth sounder. Water temperature (WT) and *Chl.a* were measured *in situ* using a portable multi-parameter probe (BBE, Germany). The water total nitrogen (WTN), total phosphorus (WTP), and ammonium nitrogen (NH_4_^+^-N) contents were determined by UV spectrophotometry. The chemical oxygen demand (COD) was determined by potassium dichromate colorimetry. All measurements were made according to the relevant standards for Water Chemistry Measurements (Huang et al., 1999).

For sediment samples, three replicate surface sediments were collected using a Peterson grab sampler, air dried at room temperature, ground, and sieved through a 2 mm mesh. The SOM, STP, and sediment total nitrogen (STN) were analyzed in the laboratory (Nelson and Sommers, 1982).

### 2.3 Data processing and analysis

All statistical analyses and plotting were performed using R 4.4.2 (R Core Team, 2023). Mean importance values were calculated as the sum of relative density (seed bank) or relative biomass (submerged macrophytes) and relative abundance. Four diversity indices were applied to characterize submerged macrophyte communities (**Supplementary Appendix S2**). A four-set Venn diagram, was used to analyze species overlap and uniqueness.

Non-metric multidimensional scaling (NMDS), based on Bray–Curtis dissimilarity matrices of Hellinger-transformed species abundance data, was applied to visualize seasonal differences in submerged macrophyte community structure, with stress values < 0.2 indicating reliable ordination results. A permutational multivariate analysis of variance (PERMANOVA) with 999 permutations was implemented to test for significant seasonal differences in community composition. β-diversity was used to quantify spatial heterogeneity in species composition, and based on the Jaccard dissimilarity index, community differences were partitioned into two core components: species replacement (Repl) and richness difference (RichDiff). A ternary plot framework was adopted to visualize the proportional contributions of Similarity, Repl, and RichDiff for each pair of sampling sites. To further quantify the relative contributions of deterministic and stochastic processes to community assembly, the modified stochasticity ratio (MSR) was calculated for each season.

Redundancy analysis (RDA) and canonical correspondence analysis (CCA) were combined with a 999-time Monte Carlo permutation test (Lai et al., 2022) to quantify the relationships between submerged macrophyte community structure and environmental factors, identify significant driving factors, and express the explanatory power of each factor as the percentage of variance explained.

A decision matrix analysis method of four-quadrant was used to explore the relationship between recovery potential and environmental pressure in submerged macrophyte communities (Lei et al., 2024). The matrix was divided into four quadrants representing combinations of high or low environmental pressure and high or low recovery potential and was used to classify site trajectories into ecological states ranging from severely degraded to reference−like. In the matrix, recovery potential (X-axis) was quantified as the mean Z-score of seed bank germination density (seeds m^-^²) and submerged macrophyte species richness (from the standing community in the same season), while environmental pressure (Y-axis) was calculated from the mean Z-score of key limiting factors identified by RDA and CCA. These are the factors that significantly constrain community structure in each season. Z-scores were calculated across all 21 sites per season.

## 3 Results

### 3.1 Seasonal variation in submerged macrophyte communities and seed banks

**Figure 2:**
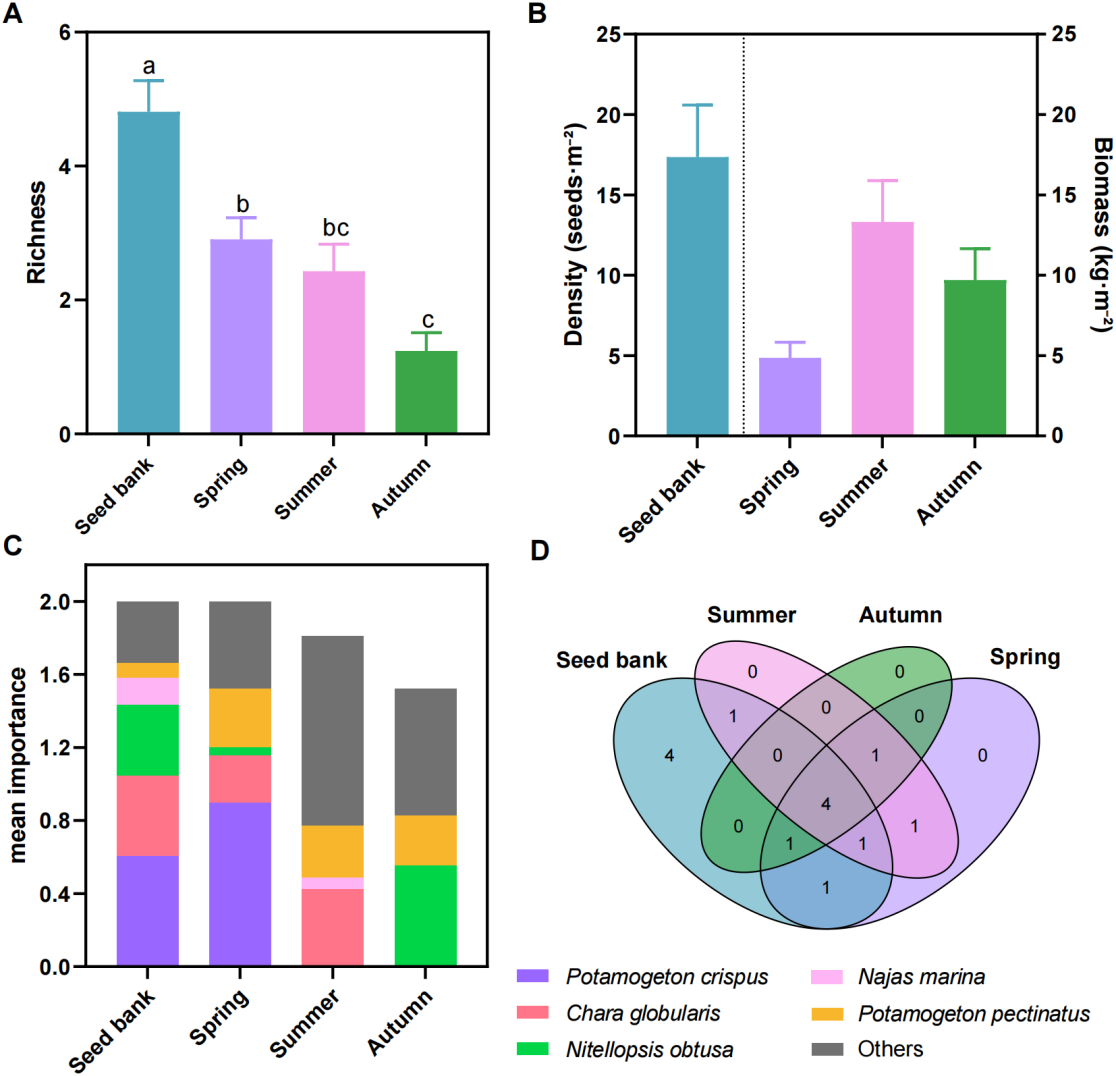
Seasonal dynamics of seed bank and submerged macrophyte communities. **(A)** species richness in the seed bank, spring, summer, and autumn submerged macrophyte communities (mean ± standard error). Different lowercase letters indicate significant differences among groups based on one-way ANOVA followed by Tukey’s HSD *post-hoc* test (*P* < 0.05). **(B)** seed bank germination density (seeds·m^-^², left axis) and total biomass of submerged macrophytes (kg·m^-^², right axis). Bars represent mean values for each group. **(C)** the proportion of the top 5 dominant species in the seed bank among the species across spring, summer, and autumn submerged macrophyte communities. **(D)** Venn diagram illustrating species distribution and overlap among seed bank and seasonal submerged macrophyte groups (numbers represent species counts).

Species richness differed markedly among the seed bank and submerged macrophytes across seasons (**Figure 2A**). The seed bank had the highest richness (mean ≈ 4.8), followed by spring submerged macrophytes (mean ≈ 3.0), summer submerged macrophytes (mean ≈ 2.9), and autumn submerged macrophytes (mean ≈ 1.4). Results from one-way ANOVA with Tukey’s HSD test revealed significant differences (*P* < 0.05). The seed bank was consistently richer than submerged macrophyte communities, whose richness declined progressively with seasonal succession.

Seed bank germination density and submerged macrophytes biomass also showed contrasting patterns (**Figure 2B**). Germination density was high in the seed bank (mean ≈ 17.5 seeds·m^-^²). Biomass of spring submerged macrophytes was low (mean ≈ 5 kg·m^-^²), increased in summer (mean ≈ 13.8 kg·m^-^²), and declined in autumn (mean ≈ 9.5 kg·m^-^²). These findings suggest that the seed bank provides a large germination pool, while submerged macrophytes biomass peaked in summer and decreased in autumn.

Dominant species composition further highlighted seasonal divergence between the seed bank and submerged macrophyte communities (**Figure 2C**). The seed bank was dominated by *Potamogeton crispus*, *Chara globularis*, *Nitellopsis obtusa*, *Najas marina*, and *Potamogeton pectinatus*. In spring submerged macrophytes, *Najas marina* was absent, but the other four species remained dominant. In summer, *Potamogeton crispus* and *Nitellopsis obtusa* were absent, and the combined proportion of *Chara globularis*, *Najas marina*, and *Potamogeton pectinatus* was below 50%. In autumn, only *Nitellopsis obtusa* and *Potamogeton pectinatus* persisted, with similarly low representation. Overall, submerged macrophyte communities diverged from the seed bank during succession, showing successive loss and reduced representation of seed bank-dominant species.

A four-set Venn diagram illustrates species uniqueness and overlap among the seed bank, spring, summer, and autumn submerged macrophytes (**Figure 2D**). The seed bank contained four unique species (*Vallisneria natans*, *Najas minor*, *Oedogonium aristatum*, *Tolypella* sp.), whereas no unique species were detected in seasonal macrophyte communities. Four species (*Ceratophyllum demersum*, *Myriophyllum spicatum*, *Hydrilla verticillata*, *Potamogeton pectinatus*) were shared by all groups. One species was shared only between the seed bank and summer macrophytes, and one species was shared by each of the seasonal pairwise combinations (spring–summer, summer–autumn, autumn–spring). No species occurred in the remaining intersections. These results indicate that the seed bank serves as the primary reservoir of unique species, whereas seasonal macrophyte communities are largely derived from the seed bank or shared among groups, with no unique species of their own. The species of submerged macrophytes and seed banks in Baiyangdian Lake are presented in **Supplementary Table 1**.

### 3.2 Seasonal changes in community structure

**Figure 3:**
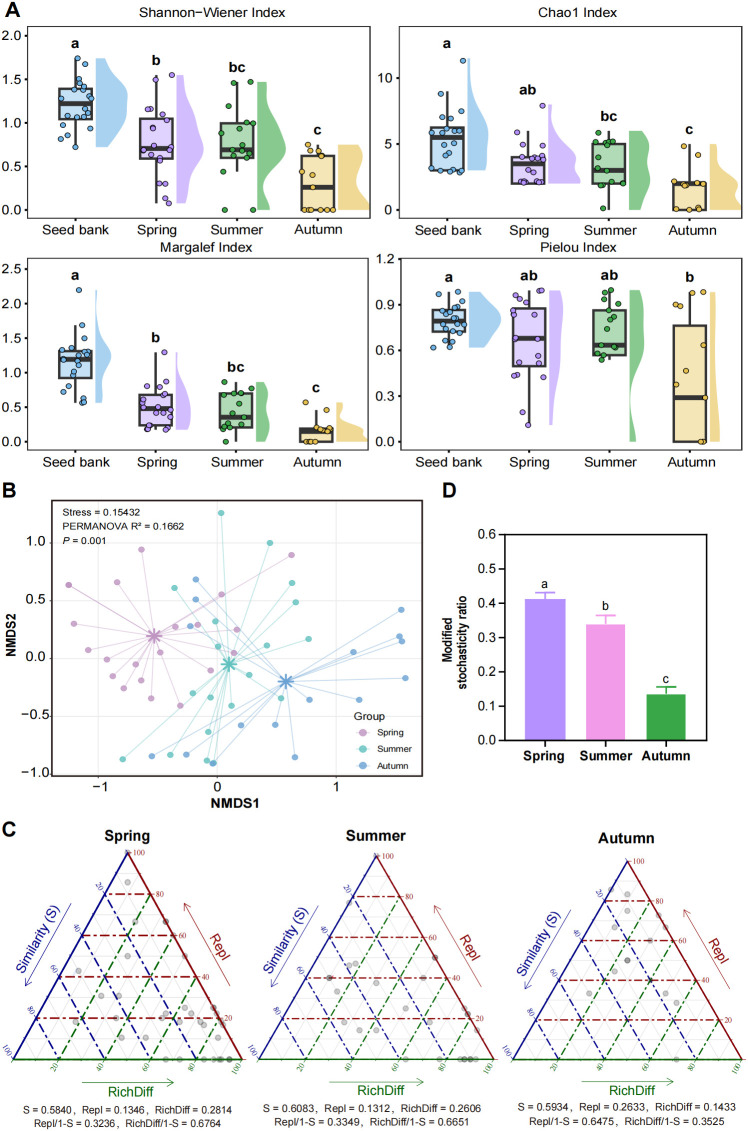
Seasonal changes in community structure and diversity of submerged macrophyte communities with their seed banks. **(A)** boxplots of alpha diversity indices; different lowercase letters indicate significant differences among groups based on one-way ANOVA followed by Tukey’s HSD *post-hoc* test (*P* < 0.05). **(B)** NMDS ordination of communities based on the Bray–Curtis distance matrix; sample points are colored by season. **(C)** Triangular plots illustrating beta diversity comparisons of submerged macrophyte communities across spring, summer, and autumn. **(D)** seasonal variations in the modified stochasticity ratio (MSR) of submerged macrophyte communities. Bars represent mean MSR values, and error bars show 95% bootstrap confidence intervals. Different lowercase letters indicate significant differences in pairwise comparisons after Kruskal-Wallis test (*P* < 0.05).

Across all diversity indices (Shannon–Wiener, Chao1, Margalef, and Pielou), the seed bank consistently exhibited significantly higher diversity than the seasonal submerged macrophyte communities (**Figure 3A**; *P* < 0.05). The Shannon–Wiener, Chao1, and Margalef indices showed a clear decreasing gradient from the seed bank to spring, summer, and autumn submerged macrophyte communities. The Pielou evenness index of the seed bank was significantly higher than that of the autumn community (*P* < 0.05), whereas no significant differences were detected between the seed bank and the spring or summer communities (*P* > 0.05). These findings indicate that the seed bank maintains higher richness and evenness, while diversity in submerged macrophyte communities declines progressively along seasonal succession.

Pronounced seasonal dynamics in community structure and diversity were evident in the submerged macrophyte communities (**Figure 3B**). NMDS ordination based on Bray–Curtis distances produced a stress value of 0.154 (< 0.2), indicating a reliable representation of community structure. PERMANOVA further confirmed significant seasonal differences in community composition (R² = 0.1662, *P* = 0.001). Sites from spring, summer, and autumn clustered distinctly, reflecting clear seasonal differentiation in community composition.

Seasonal variation induced coordinated shifts in β-diversity components and community assembly mechanisms in the submerged macrophyte communities of Baiyangdian Lake. Richness difference governed community assembly in spring and summer, whereas species replacement became dominant in autumn (**Figure 3C**). The mean MSR values with 95% confidence intervals (CIs) and significance levels (one-sample permutation test vs. 0.5) were as follows: spring = 0.412 (95% CI: 0.376–0.450, *P* < 0.05), summer = 0.340 (95% CI: 0.293–0.388, *P* < 0.05), and autumn = 0.136 (95% CI: 0.098–0.175, *P* < 0.05). All seasonal MSR values were significantly lower than 0.5, providing strong statistical evidence that deterministic environmental filtering structured seasonal community assembly. The lowest MSR value in autumn indicated the strongest filtering effect during this season (**Figure 3D**; *P* < 0.05). Collectively, these patterns underscore the role of seasonal environmental fluctuations in shaping turnover and assembly dynamics and highlight the strengthened influence of environmental filtering during autumn.

### 3.3 Key environmental drivers of seasonal shifts

Associations between submerged macrophyte communities and environmental factors in Baiyangdian Lake exhibited pronounced seasonal dynamics, with key drivers shifting across seasons. Spring communities were influenced by *Chl.a*, SD/WD, and STP; summer communities were dominated by STP with the highest explanatory power; and autumn communities were regulated by SOM, which acted as the lagged environmental filter and a key mediating factor shaping autumn community structure. STP was the only factor that remained significant in both spring and summer, whereas *Chl.a* was unique to spring and SOM was unique to autumn. These seasonal variations likely reflect dynamic shifts in environmental conditions across the lake.

**Figure 4:**
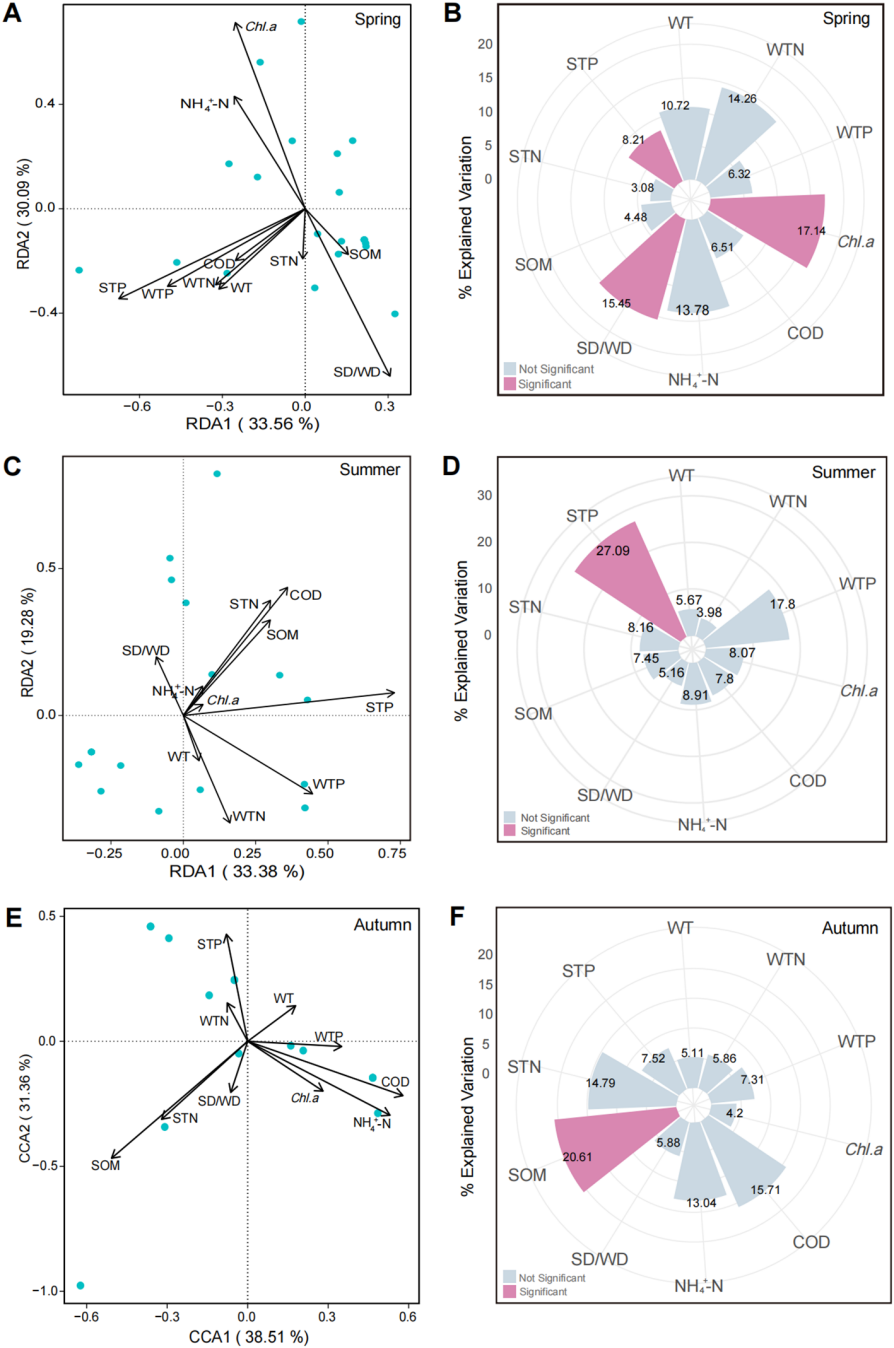
Ordination and permutation analyses of submerged macrophyte communities and environmental factors in Baiyangdian Lake across spring, summer, and autumn. **(A, B)** spring ordination and permutation tests based on RDA. **(C, D)** summer ordination and permutation tests based on RDA. **(E, F)** autumn ordination and permutation tests based on CCA.

To assess seasonal associations between submerged macrophyte communities and environmental factors, DCA was first used to select appropriate ordination methods, followed by RDA or CCA and 999-run Monte Carlo permutation tests. In spring, the maximum DCA gradient length was < 3.0, so RDA was applied; the first two axes explained 63.65% of the variation (33.56% and 30.09%; **Figure 4A**). Permutation tests identified *Chl.a* (17.14%, positive), SD/WD (15.45%, negative), and STP (8.21%, negative) as significant drivers (*P* < 0.05; **Figure 4B**). In summer, the maximum DCA gradient length was < 3.0, so RDA was used; the first two axes explained 52.66% of the variation (33.38% and 19.28%; **Figure 4C**). STP (27.09%, positive) was the only significant driver (*P* < 0.05; **Figure 4D**), whereas *Chl.a* explained only 8.07% and was not significant. In autumn, the maximum DCA gradient length exceeded 4.0 (indicating a unimodal distribution), so CCA was adopted; the first two axes explained 69.87% of the variation (38.51% and 31.36%; **Figure 4E**). Permutation tests identified SOM (20.61%, negative) as a significant driver (*P* < 0.05; **Figure 4F**).

### 3.4 Restoration potential and trajectory classification

**Figure 5:**
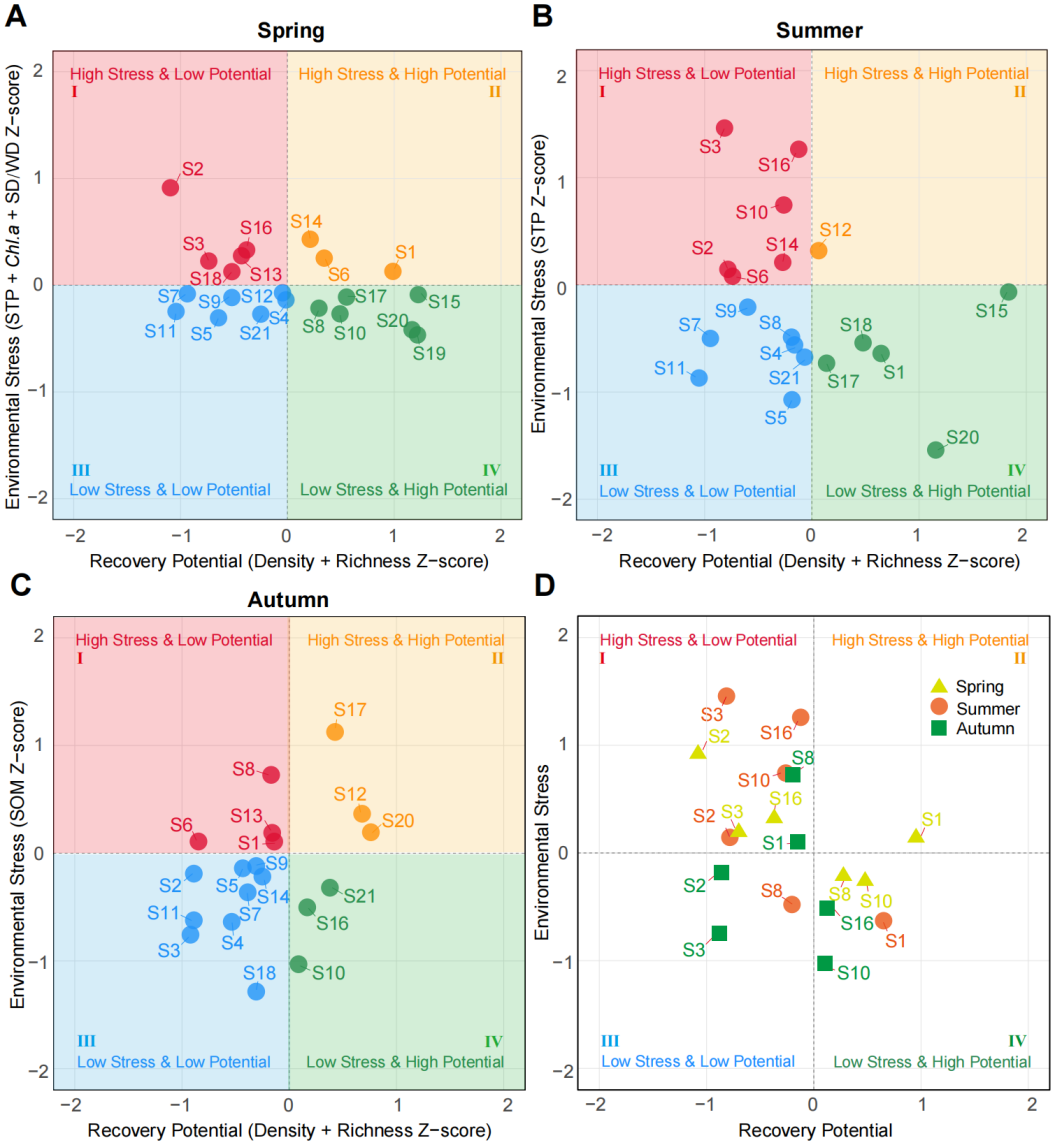
Recovery potential - environmental stress decision matrix. **(A–C)** seasonal decision matrices showing recovery potential vs. environmental pressure across 21 sites. **(D)** seasonal migration trajectories of six representative sites.

The decision matrix illustrates the relationship between recovery potential and environmental pressure across 21 sites surveyed in spring, summer, and autumn (**Figures 5A–C**). By tracking the quadrant positions of the 21 sites across the seasonal matrices, six representative sites with distinct ecological dynamics were identified, and their seasonal migration trajectories were classified into five core types (**Figure 5D**; **Supplementary Table 2**). The continuous high-risk type (S3, S2) exhibited high environmental pressure and low recovery potential in spring and summer, with only slight improvement in autumn. The spring–summer high-risk with autumn potential-improvement type (S16) showed reduced pressure and increased potential in autumn, indicating a favorable seasonal recovery window. The sustained high-pressure with seasonal potential-decline type (S1) maintained high potential in spring and summer but declined sharply in autumn, suggesting cumulative stress. The seasonal pressure-fluctuation with stable-potential type (S10) maintained high recovery potential despite seasonal fluctuations in pressure, reflecting strong ecological resistance. The spring–summer stable with autumn high-risk mutation type (S8) experienced a sudden increase in pressure and a sharp decline in potential in autumn, indicating rapid ecological deterioration. These trajectory types provide a structured framework for diagnosing seasonal ecological risks and informing site-specific restoration priorities in shallow lake ecosystems.

## 4 Discussion

### 4.1 Seasonal dynamics of submerged macrophyte communities and seed bank diversity

Our results revealed a clear seasonal decline in species richness of aboveground submerged macrophyte communities from spring to autumn, whereas the seed bank consistently maintained higher richness and contained four unique species absent from the standing vegetation (**Figure 2**). These patterns reflect seasonal variation in the contribution of seed banks to submerged macrophyte community establishment (**Supplementary Figure 1**). Meanwhile, this pattern underscores the critical role of the seed bank as a diversity reservoir for submerged macrophyte communities in shallow lakes (Grillas et al., 1993). The steady decline in aboveground richness aligns with previous findings from northern shallow lakes and can be attributed to two key ecological processes: first, intensified interspecific competition for light and nutrients as macrophyte biomass increases from spring to autumn, resulting in the exclusion of subordinate species (Chambers et al., 2008; Wu et al., 2021); second, autumnal shifts in environmental conditions, including reduced water temperature and shortened photoperiod, which suppress the growth and survival of warm−season submerged macrophyte species (Chen et al., 2022; Sun et al., 2025).

The seed bank contained four unique species. These species are likely early-successional submerged macrophytes with persistent seed banks. They represent a hidden diversity pool that may contribute to community recovery (Klupczyńska and Pawłowski, 2021). However, their seeds have not germinated to produce aboveground individuals. Several reasons may explain why these species remain absent from the standing vegetation. Phenological differences among species drive seasonal dynamics. For example, *Potamogeton crispus* is a winter-spring annual that naturally undergoes senescence under elevated summer water temperatures (Kunii, 1982). *Chara globularis* adapts to moderate-low temperatures; thus, only cold-tolerant species persist in autumn when water temperature declines sharply (Chemeris et al., 2020). Competition also contributes to species exclusion. In summer, submerged macrophyte biomass reaches its peak (**Figure 2B**). Consequently, competition for light, nutrients, and space intensifies, resulting in the exclusion of weaker or less competitive species (Chambers and Prepas, 1990). Environmental changes further filters community composition. High summer water temperatures and dynamic changes in sediment phosphorus impose strong physiological stress (Lu et al., 2021; Song et al., 2013). Meanwhile, fluctuations in water level may affect seed germination success. A decrease in water level increases light availability, thereby triggering the germination of light-demanding species in the seed bank. Conversely, an increase in water level reduces light penetration, therefore inhibiting seed germination (Zhao et al., 2021). As a result, intolerant species fail to survive (Barko and Smart, 1981). Reproductive strategies also contribute to species disappearance. *Najas marina* reproduces primarily by seeds. It completes reproduction in summer, after which maternal plants senesce. Thus, only seeds remain in the sediment, with no aboveground individuals present (Handley and Davy, 2005). The absence of these species from the standing vegetation does not indicate that they are non-viable; instead, their exclusive presence in the seed bank underscores its role as a “bet-hedging” strategy.

This finding emphasizes that the seed bank is not merely a passive repository of propagules but also an potential driver of community resilience (Han et al., 2022; Wang et al., 2014). For degraded submerged macrophyte communities, the native seed bank provides a low−disturbance, species−specific germplasm source for restoration, which is essential for maintaining the integrity of aquatic plant communities (An et al., 2022; Choi and Kim, 2022).

### 4.2 Seasonal shifts in β-diversity components and community assembly processes

β-diversity partitioning revealed a clear seasonal transition in the dominant processes shaping community variation: richness differences dominated community assembly in spring and summer, whereas species replacement emerged as the primary driver in autumn (**Figure 3C**). This seasonal shift reflects fundamental changes in the ecological processes that structure submerged macrophyte communities. We used the Index of Relative Importance (IRI) to evaluate the dominant species in the submerged macrophyte communities. Detailed calculation methods are provided in **Supplementary materials Appendix S2**. Based on the IRI results (**Supplementary Figure 2**), *Potamogeton crispus* was the absolute dominant species in spring. In summer, *Ceratophyllum demersum* became the predominant dominant species, while *Potamogeton crispus* began to decay and gradually disappeared. In autumn, the species richness of submerged macrophytes sharply declined to its lowest level of the year, with *Ceratophyllum demersum* and *Nitellopsis obtusa* becoming dominant, indicating their good adaptability to cooler conditions. In spring and summer, the rapid growth of submerged macrophytes leads to uneven colonization and establishment across sites, producing a nested community structure in which species-poor sites are subsets of species-rich sites (richness-difference dominance) (Chappuis et al., 2012; Wu et al., 2021). This pattern aligns with the habitat-filtering hypothesis, whereby sites with more favorable water clarity and nutrient conditions support higher species richness, whereas harsher sites support only a subset of tolerant species (Passy et al., 2017).

In autumn, the shift toward species-replacement dominance indicates that turnover in community composition becomes the primary source of β-diversity. This shift can be explained by two linked mechanisms: (1) intensified environmental filtering in autumn, as indicated by our assembly-process analysis, which drives species sorting along the sediment organic-matter gradient and produces distinct species compositions across environmental conditions (Holyoak et al., 2020); and (2) the senescence of summer-growing species combined with the persistence of cold-tolerant species, which generates site-specific turnover as different sites support distinct sets of late-season tolerant species (Westcott et al., 1997).

Our analysis further showed that deterministic processes dominated community assembly across all seasons, with environmental filtering being most pronounced in autumn (**Figure 3D**). This finding advances our understanding of submerged macrophyte community ecology: unlike many terrestrial plant communities, where stochastic processes often play substantial roles, submerged macrophyte communities in shallow lakes are primarily structured by deterministic environmental filtering, particularly in the late growing season (Chase, 2010; Stegen et al., 2012). In autumn, Baiyangdian Lake exhibited a marked decline in water temperature and a rising trend in SOM (**Supplementary Figures 3A, B**). Correlation analysis indicated that declining WT and SOM accumulation jointly exerted a strong environmental filtering effect. SOM was significantly negatively correlated with species richness (*R* = −0.57, *P* = 0.021; **Supplementary Figure 3C**). Although WT was not significantly correlated with richness (*R* = −0.20, *P* = 0.45; **Supplementary Figure 3D**), the pronounced post-summer temperature decline remained a key seasonal filter excluding cold-intolerant species. The synergistic effect of autumn cooling and SOM accumulation ultimately drove seasonal turnover in submerged macrophyte communities, resulting in a simplified community structure (Baert et al., 2016).

### 4.3 Key environmental drivers of community

We found that the key environmental drivers of submerged macrophyte community structure shifted markedly across seasons: *Chl.a*, SD/WD and STP shaped community structure in spring, STP was the sole significant driver in summer, and SOM emerged as the lagged environmental filter and a key mediating factor in autumn (**Figure 4**). This seasonal transition is closely linked to the phenology of submerged macrophytes and seasonal shifts in lake conditions, revealing the dynamic nature of environmental filtering in aquatic plant communities (Chambers et al., 2008; Wang et al., 2020).

In spring, the critical roles of *Chl.a* and SD/WD reflect the light limitation experienced during early submerged macrophyte growth. Spring is the key germination and colonization period for submerged macrophytes, and water clarity directly determines light availability in the water, the primary factor limiting seedling establishment (Barko and Smart, 1981; Hilt et al., 2006). Meanwhile, STP in spring provides essential nutrients for early growth, as sediment phosphorus is the primary nutrient source for rooted submerged macrophytes (Søndergaard et al., 2017). In summer, the exclusive dominance of STP indicates that nutrient availability becomes the primary limiting factor for community structure, as rapid macrophyte growth increases nutrient demand and intensifies interspecific competition for sediment phosphorus (Yang et al., 2024). The shift to SOM as the lagged environmental filter and a key mediating factor in autumn is a key finding of this study. After the peak of summer submerged macrophyte biomass, the senescence and decomposition of plants in autumn lead to the rapid accumulation of SOM in sediments. Increased SOM reduces the sediment redox potential, releases toxic sulfides, and inhibits the root growth of submerged macrophytes, further exacerbating plant senescence and community filtering (Longhi et al., 2008; Wang et al., 2017). The filtered community has a lower capacity for nutrient absorption and biomass accumulation, which in turn promotes more plant residue decomposition and SOM accumulation, forming a positive feedback loop (Bornette and Puijalon, 2011; Madsen et al., 2001).

This seasonal shift in drivers provides critical insights for the dynamic management of submerged macrophyte communities: environmental constraints on plant growth vary across seasons, and restoration strategies must align with these seasonal shifts to be effective. It should be noted that because different ordination methods (RDA vs. CCA) were applied across seasons, the explained variance percentages cannot be directly compared as absolute values. Instead, our interpretation emphasizes the identity and relative importance of significant drivers within each season. This perspective highlights how distinct environmental factors shape community structure at different times of the year, rather than focusing on numerical differences in variance explained.

### 4.4 Recovery trajectory classification and implications for submerged macrophytes conservation

Using a decision−matrix framework integrating seed bank recovery potential and environmental pressure, we classified sites into five distinct seasonal trajectory types: continuous high−risk, seasonal improvement, cumulative stress, stable−resistant, and abrupt deterioration (**Figure 5D**; **Supplementary Table 2**). Community species composition characteristics corresponding to each of the five trajectory types are shown in **Supplementary Table 3**. The five trajectory types identified in this study likely arise from interactions among seed bank robustness, sediment biogeochemistry, and cumulative seasonal stress. Sites exhibiting persistent high pressure and low recovery potential may be constrained by chronically elevated phosphorus concentrations or degraded sediment structure, both of which can suppress seed germination and hinder submerged macrophyte establishment (Wang et al., 2017; Zhou et al., 2022).

In contrast, sites maintaining stable recovery potential despite fluctuating pressure (e.g., S10) may possess dense or diverse seed banks capable of buffering short−term environmental variability. Such seed banks act as biological insurance, enabling rapid recolonization when conditions temporarily improve (Bakker et al., 2013). The abrupt autumn deterioration observed at sites such as S8 suggests a threshold−like response, where cumulative seasonal stress surpasses the system’s buffering capacity. This pattern aligns with shallow lake regime−shift theory, which posits that once sediment organic matter, internal nutrient loading, or turbidity exceed critical levels, macrophyte communities may collapse rapidly (Madsen et al., 2001; Scheffer et al., 2001). The fact that these shifts occur in autumn implies that late−season sediment processes such as internal phosphorus release may play a decisive role in driving sudden declines (Sun et al., 2022).

Differentiated ecological management strategies were developed for the five trajectory types, enabling a shift from static assessment to dynamic, precision-oriented regulation. For the spring–summer continuous high-risk type (S3, S2), the recommended strategy is targeted pollution interception and potential enhancement, focusing on controlling fixed pollution sources during high-pressure seasons and strengthening baseline recovery potential through native pioneer planting and regular monitoring of STP. For the spring–summer high-risk with autumn potential improvement type (S16), management should leverage natural recovery by protecting autumn habitat quality and identifying environmental drivers responsible for potential improvement. For the sustained high pressure with seasonal potential decline type (S1), year-round pressure control and autumn potential protection are essential. Continuous STP reduction, habitat maintenance, and supplementary measures such as native replanting and microbial enhancement can help mitigate the sharp autumn decline. For the seasonal pressure fluctuation with stable potential type (S10), seasonal precision management is appropriate. Strengthening buffer zones during summer pressure peaks and maintaining native dominant species are sufficient to preserve the consistently high recovery potential. For the spring–summer stable with autumn high-risk mutation type (S8), management should prioritize identifying mutation sources and pre-emptive protection. Investigating autumn pressure surges, establishing buffer zones, and conducting pre-autumn inspections can help prevent abrupt deterioration. Comparative analyses of species and environmental indicators can support targeted emergency planning.

Collectively, this trajectory classification provides a plant−community−based framework for targeted conservation and restoration of submerged macrophytes, aligning management actions with the natural assembly processes and seasonal dynamics of aquatic plant communities.

### 4.5 Limitations and future perspectives

This study focused on the seasonal dynamics of submerged macrophyte communities in a single growing season, and long-term multi-year monitoring is required to verify the interannual stability of the observed assembly patterns. The functional traits of submerged macrophyte species were not integrated into the assembly process analysis. Future studies should link functional trait variation to seasonal environmental filtering to further elucidate the mechanistic basis of community assembly.

In addition, because seed bank sampling was conducted only in spring (prior to the main growing season), we were unable to directly quantify the impacts of water-level fluctuations across seasons or capture seasonal dynamics of the seed bank. Moreover, vertical stratification sampling of the seed bank was not performed, preventing assessment of the contribution of the deep persistent propagule bank. Therefore, we recommend that future studies integrate hydrological monitoring with seasonal seed bank sampling and adopt stratified sampling combined with germination experiments to further elucidate the vertical distribution patterns of submerged macrophytes.

The four-quadrant framework of the decision matrix provided an initial visualization of ecological status in the study area for the first time, it shifts from static quadrant assessment to dynamic seasonal succession. However, the current trajectory classification remains limited to apparent spatiotemporal characteristics and does not provide definitive conclusions about underlying driving mechanisms. We recommend that future studies integrate field monitoring data with multivariate statistical models. This will refine mechanistic analyses and model calibration. It will also improve the scientific rigor and predictive capacity of the evaluation system.

## 5 Conclusions

In summary, this study reveals the seasonal assembly mechanisms of submerged macrophyte communities and highlights the roles of β−diversity dynamics, environmental filtering, and seed bank potential. These findings advance the understanding of aquatic plant community ecology and provide a scientific basis for the conservation and restoration of submerged macrophytes in shallow lakes. The seed bank acts as a stable reservoir of species diversity, offering an important biological safeguard that can support community recovery when the standing macrophyte community declines. Seasonal reductions in macrophyte diversity highlight periods of heightened ecological vulnerability, underscoring the need for timely management interventions. Seasonal shifts in key environmental drivers, particularly the cross-seasonal influence of STP, indicate that nutrient regulation remains a central management priority. Integrating these ecological patterns with the five identified trajectory types offers a practical framework for diagnosing seasonal ecological risks and aligning management actions with site-specific and season-specific restoration opportunities, thereby promoting more adaptive and effective shallow lake management.

## Data Availability

The original contributions presented in the study are included in the article/**Supplementary Material**, further inquiries can be directed to the corresponding authors.
