# Supplementary material for: Seed bank contributions and environmental filtering shape seasonal dynamics and restoration potential of submerged macrophytes in Baiyangdian Lake
Source: Front Plant Sci. 2026 May 8;17:1830771. doi: 10.3389/fpls.2026.1830771 (PMC13194521; doi:10.3389/fpls.2026.1830771)
Supplement: Supplementary file 1 [file DataSheet1.pdf]

Seed bank contributions and environmental filtering shape seasonal dynamics and restoration potential of submerged macrophytes in Baiyangdian Lake

Kai Mo[1], Chen Wang[1], Yu Jin[1], Zhan Shi[1], Yanzhe Tian[1], Lei Jin[1,2*], Cunqi Liu[1*]

[1]*College of Life Sciences, Hebei University, Baoding, China*

[2]*Field Scientific Observation and Research Station of Lake and Wetland Ecosystems in Baiyangdian Basin, College of Life Sciences, Hebei University, Baoding, China*

* Corresponding author

Email: liucunqi@sina.com (Cunqi Liu), hbujinlei@hbu.edu.cn (Lei Jin)

Supplementary Material

Table S1. Species of submerged macrophytes and seed banks in Baiyangdian Lake, 2024.

No.	Family	Genus	Species	Submerged macrophyte survey	Seed bank germination experiment
1	Ceratophyllaceae	*Ceratophyllum*	*C. demersum*	+	+
2	Haloragaceae	*Myriophyllum*	*M. spicatum*	+	+
3	Hydrocharitaceae	*Hydrilla*	*H. verticillata*	+	+
4		*Vallisineria*	*V. natans*	-	+
5	Lentibulariaceae	*Utricularia*	*U. vulgaris*	+	-
6	Najadaceae	*Najas*	*N. marina*	+	+
7			*N. minor*	+	+
8	Potamgetonaceae	*Potamogeton*	*P. crispus*	+	+
9			*P. pectinatus*	+	+
10			*P. malaianus*	+	-
11	Characeae	*Nitellopsis*	*N. obtuse*	+	+
12		*Chara*	*C. globularis*	+	+
13		*Lychnothamnus*	*L. barbatus*	-	+
14		*Tolypella*	*Tolypella* sp.	-	+
Total	7 Families	11 Genera	14 Species	11 Species	12 Species

Note: "+" indicates the presence of the species; "-" indicates the absence of the species.

Table S2. Five types of seasonal migration trajectories for six representative sites

Trajectory type	Site number	Spring quadrant	Summer quadrant	Autumn quadrant
Spring-summer continuous high-risk	S3、S2	I	I	Ⅲ
Spring-summer high-risk with autumn potential improvement	S16	I	I	Ⅳ
Sustained high pressure with seasonal potential decline	S1	Ⅱ	Ⅱ	I
Seasonal pressure fluctuation with stable potential	S10	Ⅳ	Ⅱ	Ⅳ
Spring-summer stable with autumn high-risk mutation	S8	Ⅳ	Ⅳ	I

Table S3. Species composition and seasonal dynamic of submerged macrophyte communities at five trajectory tpyes in Baiyangdian Lake

Site	Trajectory types	Species composition			Seasonal community dynamics
		Spring	Summer	Autumn	
S2	Continuous high-risk	*Myriophyllum spicatum*, *Potamogeton crispus*	*Ceratophyllum demersum*	No submerged macrophytes detected	No continuity of species between seasons, with early-spring species dominating in spring; original species were replaced by *Ceratophyllum demersum* in summer; all submerged macrophytes disappeared in autumn, and the community declined rapidly.
S3	Continuous high-risk	*Ceratophyllum demersum*, *Potamogeton crispus*	*Ceratophyllum demersum*	No submerged macrophytes detected	The community declined seasonally, with only 2 species in spring; *Potamogeton crispus* disappeared in summer, leaving only *Ceratophyllum demersum*; no submerged macrophytes were detected in autumn, and the community completely died out.
S16	Seasonal improvement	*Potamogeton pectinatus*, *Chara globularis*, *Potamogeton crispus*	*Chara globularis*	*Nitellopsis obtusa*	Relatively diverse species in spring, monospecific communities in both summer and autumn with complete species turnover, no inter-seasonally stable species, and rapid community structure turnover.
S1	Cumulative stress	*Potamogeton pectinatus*, *Nitellopsis obtusa*, *Ceratophyllum demersum*, *Hydrilla verticillata*, *Myriophyllum spicatum*, *Potamogeton crispus*	*Potamogeton pectinatus*, *Ceratophyllum demersum*, *Hydrilla verticillata*, *Chara globularis*, *Myriophyllum spicatum*	*Nitellopsis obtusa, Hydrilla verticillata*	The number of species decreased continuously with seasons, with the highest species richness in spring; *Nitellopsis obtusa* and *Potamogeton crispus* disappeared in summer, while *Chara globularis* newly emerged; only 2 species remained in autumn, with a greatly simplified community structure, showing a gradual recession trend.
S10	Stable-resistant	*Potamogeton pectinatus*, *Nitellopsis obtusa*, *Potamogeton malaianus*, *Potamogeton crispus*	*Potamogeton pectinatus*	*Nitellopsis obtusa*	The highest number of species in spring, only *Potamogeton pectinatus* remained in summer, replaced by *Nitellopsis obtusa* in autumn; the community structure was simple in summer and autumn, with significant species turnover.
S8	Abrupt deterioration	*Ceratophyllum demersum*, *Utricularia vulgaris*, *Myriophyllum spicatum*, *Potamogeton crispus*	*Ceratophyllum demersum*, *Hydrilla verticillata*	*Potamogeton pectinatus*	Species richness declined seasonally with complete species turnover; early-spring species dominated in spring, most early-spring species disappeared in summer, only *Potamogeton pectinatus* remained in autumn, and the community tended to be simplified.

Note: Data were compiled from seasonal species abundance from 2024 field surveys in Baiyangdian Lake.

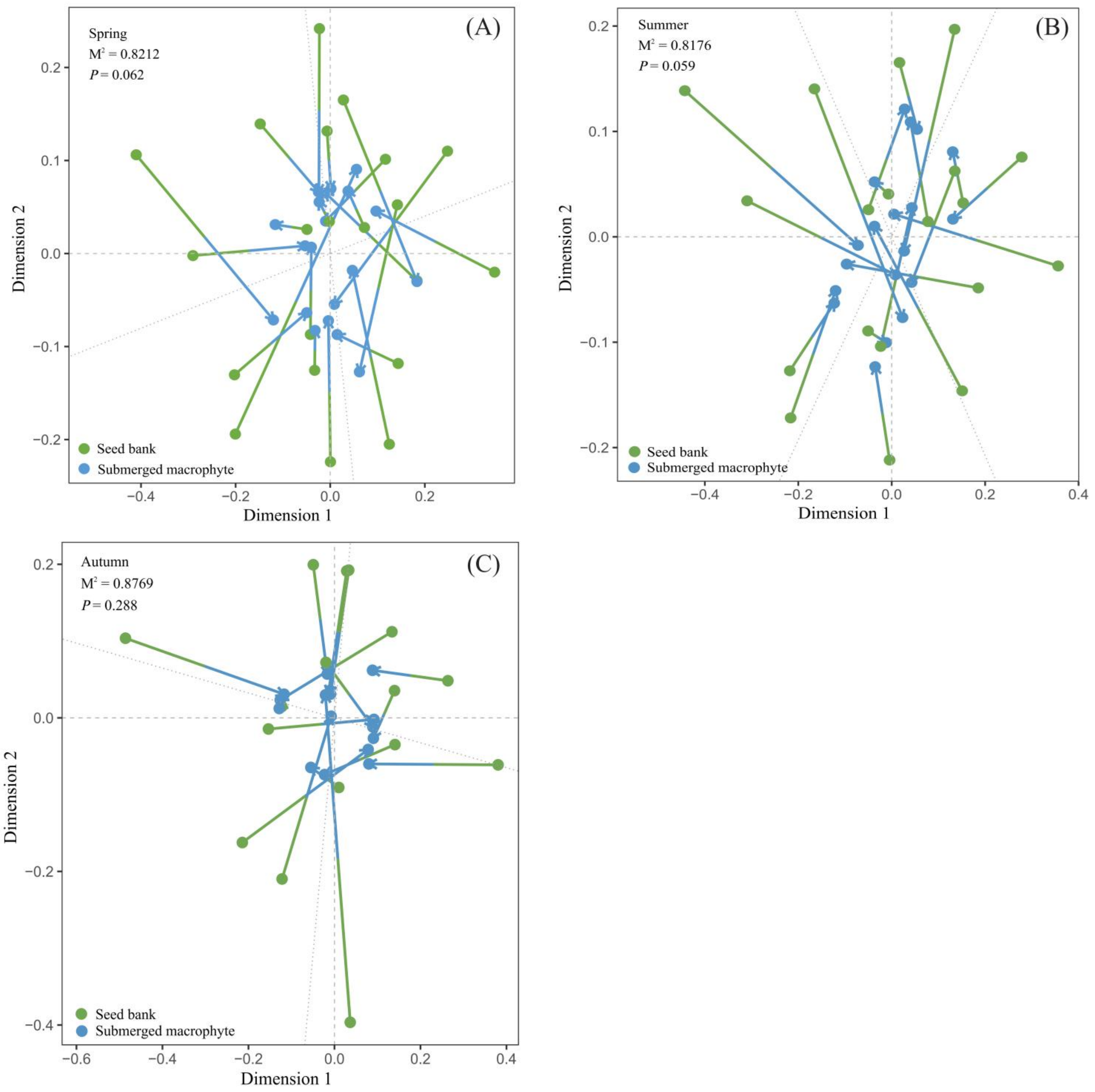

Figure S1. Procrustes ordination plots. Procrustes analysis was used to compare the configurational similarity between seed bank (green dots) and submerged macrophyte (blue dots) communities across spring (A), summer (B), and Autumn (C) in Baiyangdian Lake, with lines connecting paired samples from the same site.

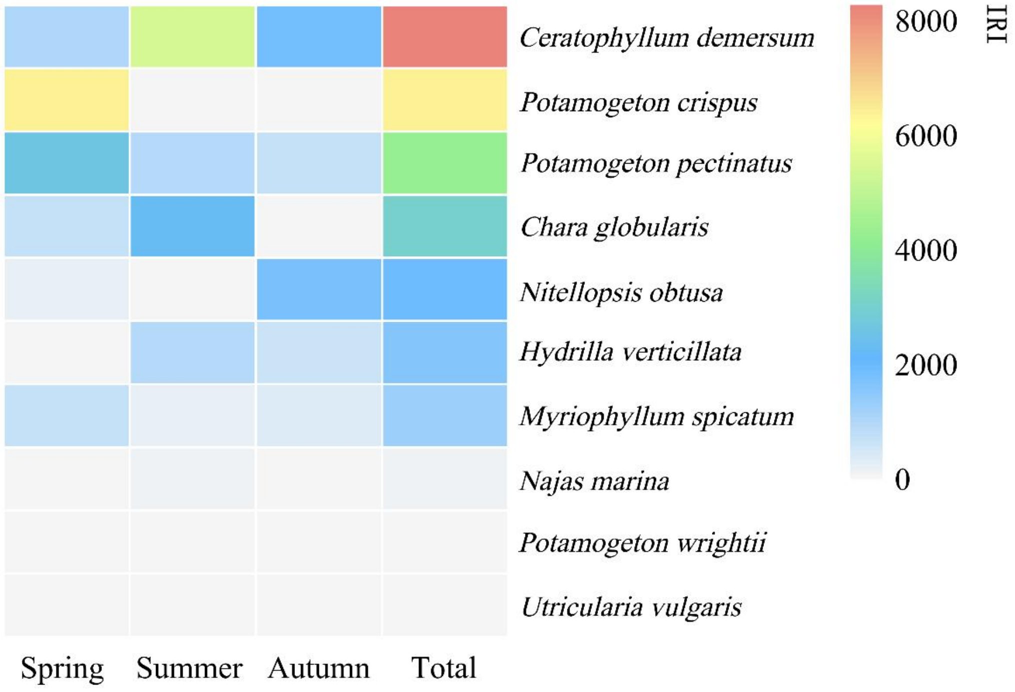

Figure S2. Dominant species of submerged macrophytes in Baiyangdian Lake across seasons.

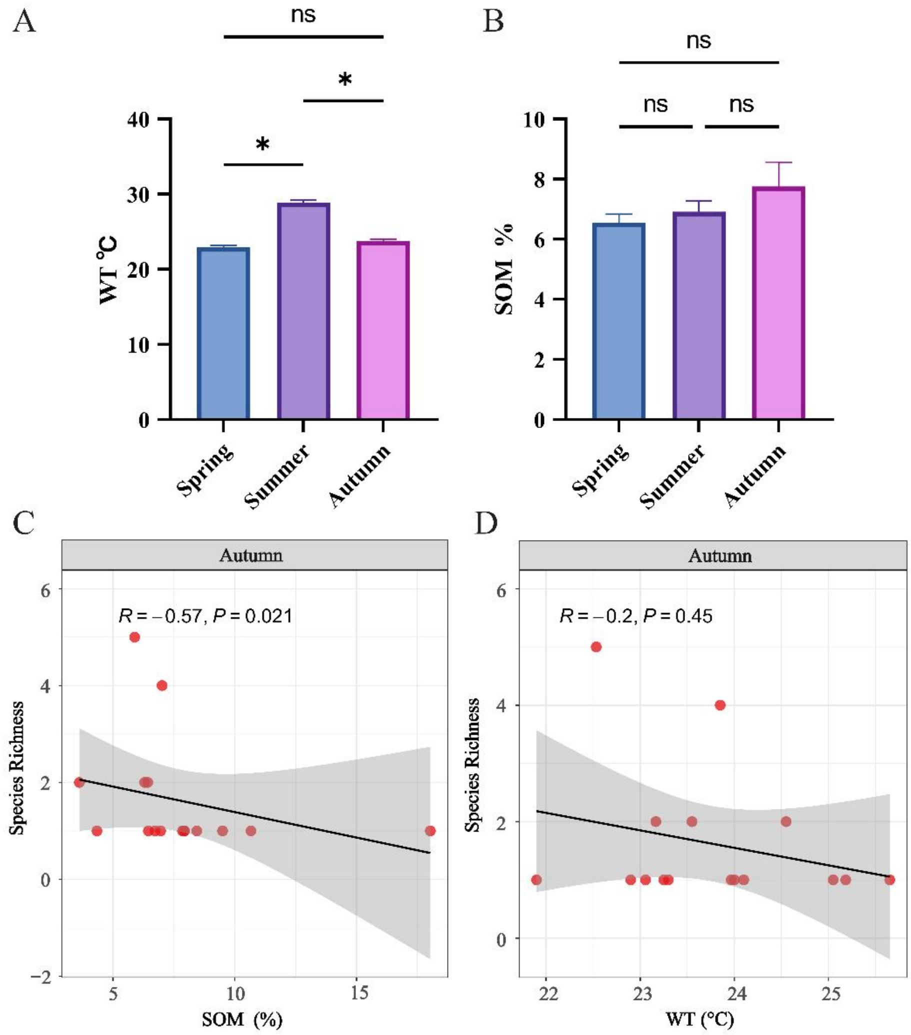

Figure S3. Seasonal patterns of water temperature (WT), sediment organic matter (SOM), and their relationships with submerged macrophyte species richness in Baiyangdian Lake.
(A) Seasonal variation in WT among spring, summer, and autumn. (B) Seasonal variation in SOM across seasons. In panels (A) and (B), bars represent the mean values ± standard error (SE). Statistical significance was determined by one-way ANOVA followed by Tukey's HSD post-hoc test; * denotes $P < 0.05$, and "ns" indicates no significant difference ($P > 0.05$). (C) Spearman correlation analysis between SOM and submerged macrophyte species richness at the site level during autumn. (D) Spearman correlation analysis between WT and submerged macrophyte species richness during autumn. In panels (C) and (D), the solid black line indicates the linear regression fit, the gray shaded area represents the 95% confidence interval (CI) of the fit, and the Spearman correlation coefficient (R) and significance level (P) are labeled in the upper left corner.

Appendix S1. Method for submerged macrophyte collection

1. Equipment preparation

Pronged grab sampler with an opening area of 0.19 m²;

GPS unit, waterproof labels, polyethylene sealable sample bags, etc.

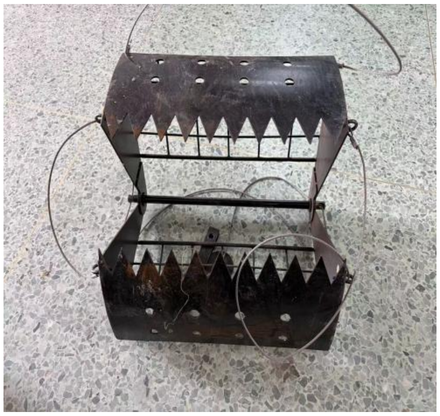

Figure A1. Pronged grab sampler

2. Plant collection

a) Each sampling site is precisely located using a handheld GPS to ensure repeated sampling at the same position across different seasons.

b) Fully open the pronged grab sampler and lower it vertically to the lake bottom. Randomly perform 3–6 grabs at each sampling site, depending on plant density: 3 grabs in sparse areas and 6 grabs in dense areas. Close the sampler to collect submerged macrophytes within the sampling site, then pull the rope to lift the sampler out of the water.

c) Place all collected plants into a field bucket filled with clean water, gently rinse to remove sediment and epiphytes, and then drain excess water. Samples from each site are placed separately into polyethylene bags labeled with the site and date, and then transported to the laboratory for further processing.

3. Laboratory processing

Remove surface water from the plants using absorbent paper and weigh the fresh mass (accurate to 0.1 g). Identify species according to a morphological taxonomic manual, and record species names and individual counts.

4. Biomass calculation

For large-scale surveys in lakes, biomass per sampling point is typically estimated by area scaling. The total biomass of submerged macrophytes at a sampling site is calculated as: Total biomass at the site = (Total wet weight of all macrophytes collected at that site) / (Single grab area × Number of grabs)

Appendix S2. Data processing and analysis

Four diversity indices were applied to characterize submerged macrophyte communities: the Shannon–Wiener index (Shannon, 1948), which integrates species richness and evenness; the Chao1 index (Chao, 1987), which accounts for rare species in assessing richness; the Margalef index (Margalef, 1958), which quantifies richness relative to community size; and the Pielou's evenness index (Pielou, 1966), which measures the evenness of species abundance distribution. The Index of Relative Importance (IRI) was used to evaluate the dominant species in the submerged macrophyte communities. The calculation formula is as follows:

$$\mathrm{IRI} = (\mathrm{n} + \mathrm{w}) \times \mathrm{f} \times 10000$$

where:

n is the proportion of the number of individuals of a given species to the total number of individuals of all species;

w is the proportion of the biomass of a given species to the total biomass of all species;

f is the proportion of the number of sites where a given species occurs to the total number of sampling sites.

Generally, species with IRI ≥ 1000 are defined as dominant species; species with 100 ≤ IRI < 1000 are defined as common species; species with 10 ≤ IRI < 100 are defined as general species; and species with IRI < 10 are defined as rare species.

The modified stochasticity ratio (MSR) was calculated to quantify the relative importance of deterministic and stochastic processes in the seasonal assembly of submerged macrophyte communities. MSR was derived using a taxa shuffle null model with 999 permutations based on Bray-Curtis dissimilarity, which randomly redistributes species occurrences while preserving species frequency and sample species richness to generate stochastically expected β-diversity (β_{null}). The observed β-diversity (β_{obs}) was calculated from the actual community data. MSR was computed as:

$$MSR = 1 - \frac{|\beta_{obs} - \beta_{null}|}{max(\beta_{obs}, \beta_{null})}$$

MSR values < 0.5 indicate deterministic process dominance (e.g., environmental filtering), whereas values > 0.5 indicate stochastic process dominance. To ensure statistical robustness, 95% bootstrap confidence intervals (CIs) were generated using 1000 resamplings for each seasonal MSR mean. One-sample permutation tests (n = 9999 permutations) were performed to examine whether seasonal MSR means significantly deviated from 0.5 (the threshold of pure stochasticity).

Reference:

Chao, A. (1987). Estimating the population size for capture-recapture data with unequal catchability. *Biometrics.* 43, 783-791. doi: 10.2307/2531532.

Margalef, R. (1958). Information theory in ecology. *Gen Syst.* 3, 36-71.

Pielou, E. C. (1966). The measurement of diversity in different types of biological collections. *J. Theor. Biol.* 13, 131-144. doi: 10.1016/0022-5193(66)90013-0.

Shannon, C. E. (1948). A mathematical theory of communication. *Bell System Technical Journal.* 27, 379-423. doi: 10.1002/j.1538-7305.1948.tb01338.x.
